# Erlotinib Resistance in Lung Cancer: Current Progress and Future Perspectives

**DOI:** 10.3389/fphar.2013.00015

**Published:** 2013-02-13

**Authors:** Joy Tang, Rasha Salama, Shirish M. Gadgeel, Fazlul H. Sarkar, Aamir Ahmad

**Affiliations:** ^1^Department of Pathology, Karmanos Cancer Institute, Wayne State University School of MedicineDetroit, MI, USA; ^2^Department of Oncology, Karmanos Cancer Institute, Wayne State University School of MedicineDetroit, MI, USA

**Keywords:** erlotinib, lung cancer, tyrosine kinase inhibitors, drug resistance, non-small cell lung cancer

## Abstract

Lung cancer is the most common cancer in the world. Despite modern advancements in surgeries, chemotherapies, and radiotherapies over the past few years, lung cancer still remains a very difficult disease to treat. This has left the death rate from lung cancer victims largely unchanged throughout the past few decades. A key cause for the high mortality rate is the drug resistance that builds up for patients being currently treated with the chemotherapeutic agents. Although certain chemotherapeutic agents may initially effectively treat lung cancer patients, there is a high probability that there will be a reoccurrence of the cancer after the patient develops resistance to the drug. Erlotinib, the epidermal growth factor receptor (EGFR)-targeting tyrosine kinase inhibitor, has been approved for localized as well as metastatic non-small cell lung cancer where it seems to be more effective in patients with EGFR mutations. Resistance to erlotinib is a common observation in clinics and this review details our current knowledge on the subject. We discuss the causes of such resistance as well as innovative research to overcome it. Evidently, new chemotherapy strategies are desperately needed in order to better treat lung cancer patients. Current research is investigating alternative treatment plans to enhance the chemotherapy that is already offered. Better insight into the molecular mechanisms behind combination therapy pathways and even single molecular pathways may help improve the efficacy of the current treatment options.

## Introduction

Lung cancer is a leading cause of cancer-related deaths and has one of the lowest survival rates among all cancers. In 2008, lung cancer was found to be the most commonly diagnosed cancer as well as the primary cause of cancer-related mortality for males worldwide and the second leading cause of cancer-related deaths for women (Jemal et al., 2011; Nurwidya et al., 2012). For the year 2012, it is estimated that lung cancer will account for 26% of all female cancer deaths and 29% of all male cancer deaths (Siegel et al., 2012). These statistics are consistent with tobacco usage in both genders. Furthermore, lung cancer is associated with poor prognosis because in many cases, the tumor is asymptomatic for a long duration of time (Saintigny and Burger, 2012). Many patients are diagnosed at a more advanced stage when surgery is no longer an option. Currently, metastasis and drug resistance are major problems that make treatment so difficult and add to the recurrence of the cancer. When non-small cell lung cancer (NSCLC) patients are treated with the most active forms of chemotherapeutic agents, their median survival period is only about 8–10 months (Ohe et al., 2007).

Drug resistance typically affects patients in one of two methods. Some patients may already have intrinsic resistance to the types of drugs offered through chemotherapy. In other cases, patients may initially be effectively treated with chemotherapy, but eventually develop more resistance over time, despite the utilization of a combination of therapeutic agents (Xiao and He, 2010). Currently, there are numerous attempts to overcome drug resistance to better the efficacy of chemotherapy. In addition to surgeries, patients may face several different treatment paths, including radiotherapy (RT) and chemotherapy. There are also different methods in treating lung cancer patients with chemotherapy, including molecular targeted therapy. Although recent research has found several combination therapies that have successfully enhanced treatment of individual lung cancer cell lines, research has yet to discover an effective treatment plan that successfully heals all lung cancer patients. Further studies on the molecular mechanisms of combination chemotherapeutic agents may help improve current therapy treatment plans.

## Small Cell Lung Cancer

Lung cancer can be broadly divided into two categories for prognostic and treatment purposes: small cell lung cancer (SCLC) and NSCLC. SCLC is a type of lung cancer that is characterized by short cell doubling time, rapid, aggressive progression, and early occurrence of blood-borne and lymph metastasis (Chen et al., 2012). It originates from neuroendocrine bronchial cells and currently accounts for approximately 15–20% of all lung cancer patients (Rodriguez and Lilenbaum, 2010). Patients with SCLC express the highest malignancy of all lung cancer types. Currently, those with SCLC initially respond well to chemotherapy and RT, but relapse is a common problem after the initial treatment. Typically, only about 2% of SCLC patients face a 5-year survival. Furthermore, drug resistance, and particularly multi-drug resistance (MDR), has become one of the main reasons for failure in SCLC patients (Chen et al., 2012). Research shows that both single factors and multiple factors combined may lead to MDR and counteract the positive, initial effects of chemotherapy.

The molecular mechanism behind MDR in SCLC involves various factors. Past research has shown that known drug resistance mechanisms include the overexpression of outer membrane proteins and lung resistance-related proteins, abnormality of the intracellular enzyme system, enhancement of the cell repair system, and dysregulation of cell apoptosis (Chen et al., 2012). Furthermore, mutation of key genes in the body may also catalyze the development of drug resistance (Roberti et al., 2006). The gene expression profiles, which consist of activation of proto-oncogenes and inactivation or loss of tumor suppressor genes, are comparatively specific for SCLC patients (Fong et al., 1999). SCLC has been divided into either classic or variant types. Classic types generally express high differentiation and sensitivity to chemotherapy while variant types express low differentiation, have a fast growth rate and respond poorly to chemotherapy.

## Non-Small Cell Lung Cancer

Non-small cell lung cancer is the most common type of lung cancer and accounts for approximately 80–85% of all patients who have been diagnosed with lung cancer (Herbst et al., 2008; Chunhacha and Chanvorachote, 2012). These lung cancer cells can again be categorized based on their histological characteristics as squamous cell carcinoma, large cell carcinoma, and adenocarcinoma (Pore et al., 2010). NSCLC spreads slower than SCLC, so many patients who are diagnosed at an earlier stage are potentially curable, though NSCLC may often relapse at other metastatic sites. Furthermore, NSCLC is generally less responsive to chemotherapy than SCLC, so that even with surgical resection at early diagnosis, approximately 50% of NSCLC patients face recurring cancers (Kelsey et al., 2006). Moreover, approximately 40–75% of stage I to stage IIIA NSCLC patients are projected to die within 5 years even with surgery (Shapiro, 2012).

Numerous chemotherapeutic agents are already being utilized to treat NSCLC patients. For example, the drug *Cis*-diamminedichloroplatinum (II; cisplatin), a platinum-based therapeutic treatment, is usually utilized to treat NSCLC and has been shown to effectively improve patient survival (Olaussen et al., 2006). However, there are also negative effects of using cisplatin, such as toxic side-effects, which include nausea, emesis, renal failure, ototoxicity, and neurotoxicity (Barabas et al., 2008; Tsang et al., 2009). Due to the toxic side-effects and failure to treat patients long-term, cisplatin is not an ideal chemotherapeutic for NSCLC patients. Recent research has explored the development and production of different anti-cancer agents that may target specific molecular pathways, which may result in lower toxicity levels and decreased possibilities of negative side-effects.

## Current Clinical Management of Lung Cancer

Although there is currently no long-term chemotherapeutic agent that effectively treats lung cancer patients, clinics offer a wide range of treatment options that still may help prolong patient life. Treatment options differ based on the histologic form of cancer, the cancer stage, and the patient’s functional ability (Collins et al., 2007). The majority of stage I through stage IIIA lung cancer patients generally choose surgery as their primary option (Beckles et al., 2003). Another popular option is preoperative chemotherapy, which has been shown to improve survival rate in patients with NSCLC. Patients who require complete resection and no preoperative chemotherapy usually invest in adjuvant chemotherapy (Skinner et al., 1991). For patients with unresectable NSCLC, RT, and chemotherapy are excellent options for treatment. Further, certain agents have been combined with the chemotherapy to enhance its effects. The antivascular endothelial growth factor agent bevacizumab, for example, when combined with chemotherapy, has resulted in increased survival rate when compared to chemotherapy treatment alone (Sandler et al., 2006).

Furthermore, treatments are also recommended based on their probability of efficacy. For first-line chemotherapy, a platinum-based two-drug combination is suggested for patients (Azzoli et al., 2009). Studies show that the drug cisplatin, when used in combination chemotherapy, is associated with improved response rates, no change in survival rate, and increased toxicity when compared with the drug carboplatin (Ardizzoni et al., 2007). Also, the aforementioned drug bevacizumab has demonstrated great potential when used in combination with carboplatin or paclitaxel in NSCLC patients. Another drug, cetuximab, which is an EGFR monoclonal antibody, has also enhanced survival when used in combination with cisplatin and vinorelbine in patients positive for EGFR expression. There are various drugs that are considered relatively effective as a first-line therapeutic, depending on the lung cancer.

The second-line chemotherapy treatment options, which are suggested after primary treatment fails to yield effective results, do differ from the first-line drugs. Approximately 30% of NSCLC patients who undergo first-line cancer treatment are candidates for second- or third-line therapy. The first agent that was approved for second-line therapeutics was docetaxel (Fossella et al., 2000). Other drugs that were also soon approved include pemetrexed, erlotinib, and gefitinib (Hanna et al., 2004; Kim et al., 2008). However, recent studies have explored an alternative non-cross resistance therapy, known as switch maintenance or continuation maintenance therapy, which can be used after first-line therapy in place of the cytotoxic chemotherapy that the aforementioned drugs fulfill (Azzoli et al., 2011). Switch maintenance aims to immediately treat patients with an alternative, single-agent chemotherapy. For example, pemetrexed would be suggested for patients with non-squamous histology and docetaxel, erlotinib, or gefitinib for unselected patients (Ciuleanu et al., 2009; Fidias et al., 2009; Cappuzzo et al., 2010; Takeda et al., 2010). Research is currently still exploring other possible second-line therapeutic options and improving current treatment plans.

Cancer pain, which can arise from local effects of the cancer, regional spread of the tumor, or from anti-cancer therapy, is one of the most common symptoms of lung cancer and also needs to be treated. Lung cancer patients experience more symptom distress than any other cancer patients (Caraceni and Portenoy, 1999; Cooley, 2000; Simmons et al., 2012). The pain is characterized into acute pain or chronic pain, which usually differ by duration of pain and predictability (Carr and Goudas, 1999). The World Health Organization (WHO) has created a series of steps to treat cancer patients for pain relief, including treatment with the anti-inflammatory drug paracetamol, weak opioids (e.g., codeine), morphine, or adjuvant analgesics (Hanks et al., 2001). If these steps fail to alleviate the pain, other interventional procedures which aim to interrupt or modify nerve conduction are also available.

## Epidermal Growth Factor Receptor

The epidermal growth factor receptor (EGFR) is a tyrosine kinase that contributes to the regulation of cellular homeostasis (Cohen, 1965; Nicholson et al., 2001; Wheeler et al., 2010). It is a 170 kDa membrane protein that stimulates downstream cell signaling cascades that could influence migration, apoptosis, cell proliferation, survival, and tumorigenesis (Hynes and Lane, 2005). The EGFR family consists of four members, including HER1/ErbB1, HER2/ErbB2, HER3/ErbB3, and HER4/ErbB4 (Giaccone and Wang, 2011). EGFR has been implicated in the growth of several human epithelial malignancies, including lung cancer. It is overexpressed in several cancers, including approximately 40–80% of NSCLC, which made EGFR a popular target for new drug treatment exploration (Salomon et al., 1995; Rusch et al., 1997; Brabender et al., 2001). Thus, targeting EGFR has been a fairly recent topic of research for cancer therapeutics, with a resultant series of potential molecular inhibitors that may be pursued in clinical oncology. The difficulties with EGFR inhibitor therapy also deals with drug resistance and longevity of chemotherapy. The connection between EGFR alteration and tumorigenesis makes it an ideal contender for molecularly targeted therapy (Wu et al., 2012). A better understanding of the molecular mechanisms behind EGFR inhibitors and drug treatment resistance would greatly improve the current chemotherapies offered.

## EGFR Mutations

Studies have identified some EGFR mutations in the tyrosine kinase domain in NSCLC patients which may help predict therapeutic results with tyrosine kinase inhibitors (TKIs), erlotinib, and gefitinib (Paez et al., 2004; Pao et al., 2004). These mutations, including in-frame deletion of several amino acids, resulted in tumors that became significantly more responsive to treatment by erlotinib and gefitinib than tumors that did not have EGFR mutations. These studies have advanced our understanding of the role of EGFR mutations in interacting with TKIs, but currently there is not enough research to form a reliable antibody-based EGFR therapy (Mukohara et al., 2005).

Approximately half of the NSCLC tumors found in patients who initially responded to first-generation EGFR TKIs and then develop resistance have another mutation, known as the T790M point mutation in *EGFR* (Sharma et al., 2007; Suda et al., 2009). The T790M mutation could also initially combine with erlotinib or gefitinib in the body and also contribute to initial drug resistance. Furthermore, when treated with first-generation TKIs, the T790M cells are expressed with an increasingly larger percentage of the tumor mass over time (Inukai et al., 2006). The mutation may also aid in faster tumor growth, especially when occurring in concurrence with another *EGFR*-activating mutation. In addition, other *EGFR* mutations have also demonstrated contribution to the development of drug resistance to erlotinib and gefitinib, including secondary *EGFR* kinase mutations, L858R mutations, and secondary D761Y point mutations. Better understanding of the molecular mechanisms behind EGFR mutations may benefit the use of TKIs for chemotherapy in the future.

## Erlotinib

The introduction of EGFR inhibitors, such as erlotinib, has been an important advancement in targeted chemotherapy. Erlotinib is an EGFR-specific TKI that has been approved by the Food and Drug Administration to be used as a molecularly targeted drug for lung cancer patients (Sierra et al., 2010; Diep et al., 2011; Kosaka et al., 2011). Erlotinib functions by reversibly inhibiting EGFR through competitive binding at the ATP site in the tyrosine kinase domain, which results in fewer downstream proliferative signaling pathways (Steins et al., 2010; Nguyen and Neal, 2012). It has been shown to be effective in patients whose tumors express EGFR mutations (Shepherd et al., 2005). The overexpression of EGFR in tumors has been linked to poor prognosis due to its association with tumor progression, angiogenesis, migration, and metastasis (Yarden, 2001; Mehta, 2012). Erlotinib is highly utilized in cancer therapy due to its relatively few side-effects and high efficacy in patient responders of this TKI (Lee and Wu, 2012). Initially, erlotinib was approved in 2004 as monotherapy for patients with NSCLC, and then in 2005, it was approved as for combination chemotherapy with gemcitabine (Wheeler et al., 2010). However, erlotinib has been shown to be ineffective for the majority of lung cancer patients because the patients are either initially resistant to the inhibitor or eventually develop resistance. Statistics show that approximately 10–14 months after the primary treatment of erlotinib, NSCLC patients start to develop resistance to the agent, which results in reoccurring lung cancer (Kosaka et al., 2011; Oxnard et al., 2011). In order to better treat patients with NSCLC, further studies should explore the molecular mechanisms behind erlotinib resistance development.

## Erlotinib Resistance

Epidermal growth factor receptor TKIs, such as erlotinib, are commonly utilized as part of cancer therapy. However, drug resistance is a great problem in sustaining the efficacy of such drugs. Recent studies have revealed that there is a strong link between the mesenchymal-to-epithelial transition factor (MET) activation and amplification and resistance to TKIs (Gusenbauer et al., 2012). MET, which is a proto-oncogene that encodes the receptor for the hepatocyte growth factor (HGF), is associated with inducing cell invasion and metastasis (Birchmeier et al., 2003). MET is also usually expressed along with EGFR in several human cancers, including lung cancer (Weinberger et al., 2005). HGF has been shown to activate Met and stimulate short-term resistance to EGFR TKIs. After examining the function of HGF by treating cancer tissues with it, which resulted in completely blocked EGFR tyrosine kinase activity (Weinberger et al., 2005). Thus, HGF-induced inhibition of EGFR TKIs is shown to be a common occurrence in human cancers.

The acquisition of epithelial-to-mesenchymal transition (EMT) is associated with increased cell invasion, migration, and proliferation and loss of cell adhesion proteins in numerous cancer cell lines and linked to the progression of cancer malignancies (Maitah et al., 2011). EMT is observed in many epithelial cancers, including NSCLC. Research suggests that EMT markers may be linked to EGFR inhibitors. A recent study that examined both epithelial and mesenchymal cell sensitivity to erlotinib found that mesenchymal cells exhibited increased resistance to the EGFR inhibitor in comparison to epithelial cells (Byers et al., 2013). Furthermore, this study was able to predict erlotinib sensitivity in *EGFR*-mutant and *EGFR*-wild type NSCLC based on EMT score. *EGFR*-wild type patients who showed progress from treatment with EGFR TKIs were effectively identified in several clinical studies. More importantly, it was revealed that the Axl inhibitor SGI-7079, which is expressed highly in mesenchymal cells, could reverse erlotinib resistance in mesenchymal cell lines when used in combination with erlotinib (Byers et al., 2013). This breakthrough may help improve the longevity of cancer treatment with erlotinib.

Researchers have been exploring more innovative methods to battle erlotinib drug resistance. For example, recent studies have shown that the manner in which patients are treated with erlotinib affects the efficacy and longevity of the therapy. It was revealed that high-dose pulses with low-dose continuous treatment with erlotinib impeded the development of resistance in both cases of pre- and post-emergence of resistance (Foo et al., 2012). Drug resistance has a higher probability of developing in fast drug metabolizers, which indicates that there is a potential mechanism that influences the development of drug resistance in patients. The results of this study should be further explored to enhance current kinase inhibitor knowledge and improve dosing strategies.

Another associated cause of drug resistance may be due to the methylation of death-associated protein kinase (DAPK). Emerging studies have linked epigenetic changes, including DNA methylation at CpG islands, to the development of drug resistance to many anti-cancer drugs (Ogawa et al., 2012). Evidence also indicates that genes that are differentially methylate are potential biomarker candidates. A recent study found that the DAPK is hypermethylated in drug-resistance derivatives. Moreover, restoration of DAPK in drug resistance NSCLC cells resulted in the re-sensitization of cells to therapeutic agents, including erlotinib and cetuximab (Ogawa et al., 2012). The results suggest that DAPK may play an important function in erlotinib resistance. It would be beneficial to further explore gene silencing through promoter methylation.

## Combination Therapy to Improve Treatment with Erlotinib

A recent study investigated the efficacy of a low-dose combination treatment of erlotinib and cisplatin in lung adenocarcinoma to better comprehend the role of autophagy in erlotinib resistance (Lee and Wu, 2012). Exploring the relationship between drug resistance and cell autophagy is important because autophagy functions in cell protein homeostasis and degradation of injured cellular organelles (Hsieh et al., 2009). Thus, impaired autophagy is associated with several human diseases, such as cancer, but the exact role it plays in cancer resistance remains uncertain (Shimizu et al., 2012). The result demonstrated that a low-dose erlotinib-cisplatin combination was able to overcome erlotinib resistance and also stimulate synergistic cell death. The treatment also exhibited low toxicity to the sample cells. Furthermore, the study found that altering the level of autophagy affected the cell’s sensitivity to erlotinib. Although researchers had success in overcoming drug resistance in a single cell line, this does not suggest that the treatment will be effective for all NSCLC patients. In many cases, combinations of chemotherapy and anti-cancer agents have been used for chemotherapy. However, the treatments are not effective for all other differing cell lines. The current research is an effective start to exploring the molecular mechanisms behind drug resistance and cell autophagy. Further exploration of this idea may yield more insight into a generalized drug combination that may effectively treat all lung cancer patients.

Another study demonstrated how other combination therapies may improve the current erlotinib treatment. The study found that the ATP non-competitive CDK-2/cyclin A inhibitor NBI1 will help sensitize tumor cells that have developed resistance to erlotinib to combination therapy for cell apoptosis (Orzaez et al., 2012). In addition, for tumor cells that were sensitive to erlotinib, the effective dose of erlotinib was lower when the inhibitor NBI1 was present. Combining TAT-NBI1 with erlotininb reduces the lethal dose of erlotinib in the erlotinib-sensitive cells and it also tumor cells that are resistant to erlotinib to apoptosis. This study emphasized the value in researching the therapeutic effects of EGFR inhibitors when utilized in conjunction with ATP non-competitive CDK-2/cyclin A inhibitors. This study focused on the NBI1 inhibitor, but further exploration of other CDK-2/cyclin A inhibitors may also prove valuable to the chemotherapeutic effects of erlotinib treatment.

In addition to the combination therapy of CDK-2/cyclin A inhibitors, another study examined the effect of the histone deacetylase (HDAC) inhibitor trichostatin A (TSA) to enhance the effects of erlotinib (Zhang et al., 2012). HDAC inhibitors are anti-cancer agents with the potential to enhance treatment of human cancers and TSA is a specific inhibitor of HDAC that can result in cell growth arrest. This study investigated the combining effect of TSA with erlotinib in A549 lung cancer cells. Results demonstrated that molecular targeted chemotherapy with this combination of agents led to a considerable increase in cleaved caspase-3 expression, which resulted in decreased expression of EGFR. This research provides *in vitro* support that TSA can inhibit cancer growth by contributing to the stimulation of cell apoptosis. Further research on the mechanism behind this pathway is encouraged for better understanding of this combination therapy and its effects on lung cancer.

A study that observed the effect of erlotinib combined with pemetrexed also yielded encouraging results. Both erlotinib and pemetrexed are currently approved for second-line treatment for advanced NSCLC patients. Pemetrexed is a multi-targeting antifolate cytotoxic agent that generally inhibits several enzymes associated with the folate metabolism, including thymidylate synthase, dihydrofolate reductase, and glycinamide ribonucleotide formyl transferase (Hanauske et al., 2001). When cancer cells are treated with pemetrexed, they are arrested in cell growth and then undergo cell apoptosis. The phase I study explored the treatment of lung cancer cells with a combination of erlotinib and permetrexed, which was well-tolerated and fairly successful in antitumor efficacy (Minami et al., 2012). More research on the effect of combination therapy with these two agents is undergoing phase II study.

More research on combinational therapy explored the possibility of therapy with erlotinib combined with enzastaurin. Enzastaurin, which is an oral serine/threonine kinase inhibitor that targets the protein kinase C (PKC) and phosphatidylinositol-3-kinase (PI3K) pathways, affects signal transduction linked to angiogenesis, apoptosis, and cell proliferation (Partovian and Simons, 2004). A current study that explored the treatment of patients with advanced stage IIIB or IV NSCLC with erlotinib and enzastaurin. Results revealed that while the enzastaurin did not help improve the patient response to chemotherapy, it did not worsen usual results either (Clement-Duchene et al., 2012). Overall, the combination therapy presented to be well tolerated in the NSCLC patients. This is a common outcome in combination therapy tests. Although several combinations of therapeutic agents may not improve patient outcome, in many cases, the combination therapy is simply well tolerated and does not improve or worsen patients who are treated singularly with erlotinib. Another example of this situation is observed in a study of combination therapy with erlotinib and tivantinib. Tivantinib is a selective oral, non-ATP-competitive inhibitor of the MET receptor tyrosine kinase (Goldman et al., 2012). Since the MET gene is associated with inducing tumor resistance to EGFR inhibition, tivantinib seems to have a promising role in overcoming drug resistance. However, the result of treating NSCLC patients with tivantinib and erlotinib resulted in well tolerated clinical activity. Patients did not experience significantly worse or better outcomes.

Another potential combination of therapeutic agents is treating NSCLC patients with erlotinib and cetuximab. Cetuximab is a monoclonal antibody that directs against the extracellular domain of EGFR. It functions in inhibiting phosphorylation and stimulates internalization. A recent study demonstrated that using a combination of erlotinib and cetuximab reversed the drug resistance in NSCLC in T790M and L858R mutation lung cancer cell lines (Wang et al., 2012). When compared to single treatment with erlotinib, the combination of agents led to increased apoptosis of EGFR TKI-resistance cells, decreased cell proliferation, and increased inhibition of EGFR-dependent signaling. This combination of drugs shows great potential in helping to mediate the growing drug resistance problem.

In addition to its combination treatment for chemotherapy, erlotinib has also been considered as an option in combination with RT. A recent study explored the effects of erlotinib combined with RT in numerous cancer types, including lung cancer. Since erlotinib contributes to the disruption of cell growth pathways and stimulates cell sensitivity to the effects of RT, it was predicted that combination therapy would be effective in treating lung cancer (Chinnaiyan et al., 2005; Baumann et al., 2007). It was also predicted that RT would help enhance the efficacy of erlotinib by cytoreducing tumors and creating a hypoxic environment (Tortora et al., 2007). The study reported that combining erlotinib and RT yielded moderate improvements and toxicities have been manageable (Mehta, 2012). Trials with this combination of therapy is now undergoing phase III trials, which should hopefully yield new insight into pairing erlotinib with other forms of cancer therapy.

## Overcoming Drug Resistance

### Resistance to EGFR inhibitors

Recent research is investigating a more resistive form of inhibitors to use for chemotherapy. Many studies are focusing on irreversible inhibitors that will concurrently target multiple members of the EGFR family (Giaccone and Wang, 2011). The first-generation erlotinib binds to the catalytic site of the EGFR tyrosine kinase domain through competitive binding with ATP (Suda et al., 2009). Thus, the irreversible binding mechanism of the next-generation erlotinibs, and other TKIs, is expected to increase the effectiveness of the inhibitors by prolonging the inhibition of EGFR signaling and decreasing developments to resistance. It is predicted that the irreversible inhibitor will covalently bind to the EGFR and no longer function in a competitive, reversible bind with ATP (Yun et al., 2008).

The realization that other EGFR family members, specially HER2, compensate for EGFR-targeted therapies, led to the development of irreversible inhibitors such as BIBW 2992/afatinib (Doebele et al., 2010; Spicer and Rudman, 2010; Kwak, 2011), HKI-272 (Doebele et al., 2010), and PF00299804 (Ercan et al., 2010; Kwak, 2011). Clinically, it is observed that almost all the NSCLC patients who initially show signs of disease stabilization through EGFR TKI, finally progress to secondary resistance (Batus et al., 2010). For such patients, irreversible EGFR TKIs hold a lot of promise. The irreversible TKI BIBW 2992 (afatinib) is currently being investigated in advanced clinical trials (Yap et al., 2010; Murakami et al., 2012). Another novel irreversible pan-HER inhibitor HM781-36B has recently shown promise in the inhibition of EGFR, HER2, and HER4 as well as against the gatekeeper mutation – EGFR T790M that is responsible for acquired drug resistance in lung cancer patients (Cha et al., 2012).

### Drug resistance-reversing agents

A developing strategy to counteract drug resistance progression in SCLC is to target the mitochondrial apoptosis pathway. An example is the anti-apoptotic gene Bcl-2 which is commonly overexpressed in SCLC cells and tissues (Hann et al., 2008). This is also associated with the apoptosis escape of tumor cells and chemoresistance (Rudin et al., 2004). In addition, another study found that in a phase I clinical trial, the Bcl-2 targeted inhibitor Oblimersen relieved approximately 86% of untreated SCLC patients (Oltersdorf et al., 2005). The Bcl-2 targeted inhibitors are divided into two groups: antisense oligonucleotides and small molecular weight inhibitors.

### New chemotherapeutic agents

Several new chemotherapeutic agents have been developed that are predicted to increase the success of chemotherapy. Such drug treatments include amrubicin, SABA, picoplatin, belotecan, and vinflunine (Chen et al., 2012). Amrubicin, for example, is a synthetic anthracycline that aids in blocking NDA repair by inhibiting Topoisomerase I. Already, clinical trials have resulted in increased response rates as high as 88% from combined utilization of amrubicin and picoplatin, which is a platinum analog that functions in overcoming platinum drug resistance (Tang et al., 2011). The median survival period is also recorded to be 13.6 months among SCLC patients. Furthermore, phase II clinical trials have also demonstrated the beneficial therapeutic activity of amrubicin and picoplatin in relapsed SCLC patients (Jeong et al., 2010). A new experimental treatment has recently been explored. Picoplatin, which is a new platinum compound created to surmount other platinum agents, has already exhibited decreased neurotoxicity and nephrotoxicity than other platinum compounds (Nair et al., 2011). However, the study reported that the picoplatin did not exhibit any improvements in overall survival rate when compared to other chemotherapeutic agents (Eckardt et al., 2009). Further exploration of new chemotherapeutic agents may prove to be very beneficial to helping SCLC patients overcome drug resistance.

### Targeted therapies

Better understanding of the molecular biology behind various SCLC treatment pathways has yielded new, promising targets. Recently, novel molecularly targeted drug treatments have been developed, including matrix metalloproteinase (MMP) inhibitors, thalidomide, biological vaccines, and small molecular weight inhibitors directed at receptor protein tyrosine kinases. Many of these have been tested in phase II clinical trials for success in overcoming drug resistance (Chen et al., 2012). However, most of these aforementioned treatments have failed thus far in demonstrating efficacy in clinics (Dy and Adjei, 2002; Antonia et al., 2006; Khanzada et al., 2006; Lee et al., 2008; William and Glisson, 2011). Another treatment in particular, of which efficacy is pending, is the use of simvastatin or pravastatin. Earlier studies found that simvastatin can inhibit SCLC growth, induct tumor cell apoptosis, and increase the sensitivity of SCLC to etoposide (Weiss et al., 1999). Pravastatin yields similar inhibitory effects on SCLC, and phase III clinical trials are still in effect.

### MicroRNAs

MicroRNAs (miRNAs) are small regulatory RNA molecules that post-transcriptionally regulate gene expression by targeting messenger RNAs (mRNAs; Croce and Calin, 2005; Fabian and Sonenberg, 2012). miRNAs play important roles in many cellular processes, including tumorigenesis. They are involved in many pathways associated with lung cancer. For example, ErbB2, ErbB3, and ErbB4 are all part of a family of tyrosine kinase receptors whose overexpression results in tumorigenesis and cancer cell proliferation (Holbro et al., 2003; Kiran and Deepika, 2012). Furthermore, miRNAs are also associated with the regulation of EGFR in lung cancer. The inhibition of miR-128, for example, led to upregulation of EGFR expression in an EGFR-expressing NSCLC cell line (Weiss et al., 2008). In addition, inhibition of miR-21 resulted in increased anti-apoptotic potential of an anti-EGFR tyrosine kinase inhibitor in an EGFR-mutant lung adenocarcinoma cell line (Seike et al., 2009). Another example can be seen in miR-29, which is downregulated in several human cancers, including lung cancer. miR-29 regulates epigenetic DNA methylation, which suggests that enhancing expression of this miRNA may effectively function as a therapeutic agent (Fabbri et al., 2007). In addition to the aforementioned miRNAs, studies have demonstrated the association between numerous other miRNAs and lung cancer therapy. Further investigation of the molecular mechanisms of other miRNAs may provide better insight into cancer therapeutics and how to utilize miRNAs to enhance therapy.

## Conclusions

Cancer research has progressed rapidly and led to the development of several methods of treatment, including molecular targeted chemotherapy. Although several chemotherapeutic agents have proven to be quite effective in treating numerous patients with lung cancer, in the majority of cases, patients either already were resistant to the effects of the drug or eventually developed resistance as time progressed. Erlotinib, for example, typically is effective on patients with EGFR mutations, but generally does not successfully treat lung cancer patients without malignancy reoccurrence. Although numerous mechanisms for drug resistance have been developed, they are not able to effectively account for all cases of drug resistance. However, research has shown promising potential in targeting multiple tumorigenic pathways simultaneously. Combination therapy with erlotinib has already demonstrated success in numerous lung cancer cell lines. Antitumor effects have already been observed in several studies. Current studies are exploring new agents to enhance chemotherapy and focusing on identifying potential predictors of treatment benefit (Leighl, 2012). Further investigation of these pathways may improve our knowledge of the antitumor efficacy and longevity, which will hopefully improve treatment options for lung cancer patients.

## Conflict of Interest Statement

The authors declare that the research was conducted in the absence of any commercial or financial relationships that could be construed as a potential conflict of interest.
